# Enhanced expression of miR-889 forecasts an unfavorable prognosis and facilitates cell progression in hepatocellular carcinoma

**DOI:** 10.1186/s13000-021-01111-5

**Published:** 2021-06-11

**Authors:** He Wang, Huiwen Wang, Wenyu Cui, Qiao Zhang, Jing Li, Qi Zhang

**Affiliations:** 1 Department of Interventional, Harbin Medical University Cancer Hospital, No.150 Haping Road, Harbin, Heilongjiang 150081 Harbin, China; 2 School of Pharmacy, Harbin University of Commerce, 138 Tongda Street, Harbin 150076 Heilongjiang, China

**Keywords:** MiR-889, Hepatocellular carcinoma, Prognosis, Proliferation, Migration, Invasion

## Abstract

**Background:**

As a new type of molecular marker, microRNAs (miRNAs) can be used for early diagnosis and prognosis prediction of malignant tumors, and has broad clinical application prospects. This paper mainly studies the important role of miR-889 in the occurrence and development of hepatocellular carcinoma and the prognostic significance of miR-889 in hepatocellular carcinoma.

**Methods:**

Quantitative real-time PCR analysis detected the expression levels of miR-889 in hepatocellular carcinoma tissues and cell lines. Kaplan-Meier curve and Cox regression analysis were used to explore the prognostic significance of miR-889 in hepatocellular carcinoma. The CCK-8 and Transwell assays assay were used to assess cell proliferation, migration, and invasion abilities ability.

**Results:**

The expression of miR-889 in hepatocellular carcinoma tissues was significantly higher than that in adjacent tissues. Overexpression of miR-889 was significantly associated with TNM stage, hepatitis B virus infection, and cirrhosis. Patients with high miR-889 expression had shorter overall survival than those with low miR-889 expression. And functional studies in two hepatocellular carcinoma cell lines have shown that overexpression of miR-889 significantly promoted cell proliferation, migration, and invasion *in vitro*.

**Conclusions:**

Overall, miR-889 was upregulated in hepatocellular carcinoma tissues and cell lines, and overexpression of miR-889 promoted cell proliferation, migration, and invasion in hepatocellular carcinoma cells. Based on our findings, high expression of miR-889 may promote the progression of hepatocellular carcinoma, and high expression of miR-889 is also forecasted for an unfavorable prognosis in hepatocellular carcinoma.

## Introduction

Hepatocellular carcinoma (HCC) is the most common primary liver tumor, and its incidence ranks fifth among malignant tumors and third among deaths caused by tumors [1, 2]. The occurrence of liver cancer is closely related to chronic inflammation of the liver. It is currently believed that viral hepatitis, excessive alcohol consumption, and non-alcoholic liver steatosis are important causes of liver cancer [3, 4]. The occurrence and development of liver cancer are closely related to the repeated damage and proliferation of liver cells caused by chronic inflammation of the liver [5]. The molecular mechanisms mainly include the activation of oncogenes in liver cells, the inactivation of tumor suppressor genes, and the excessive activation of signal pathways related to liver cancer [6, 7]. The molecular mechanism research on the occurrence and development of liver cancer and the translational medicine research closely integrated with the clinic will propose new intervention directions for the prevention, control, and treatment of liver cancer [8, 9].

miRNA is a type of small non-coding RNA with a length of about 18–25 nucleotides. It can participate in various physiological and pathological processes of the body by regulating the expression of target genes [10, 11]. In mammals, the mechanism of miRNA is mainly thought to inhibit the expression of target genes by acting on the 3’untranslated region of the target mRNA [12, 13]. In recent years, research on the role and mechanism of miRNA in specific physiological and pathological processes has progressed rapidly [14]. Especially in the process of tumorigenesis and development, miRNA has been proven to be a potential target for early diagnosis, prognostic judgment, and intervention treatment of tumors [15]. Many recent studies have shown the relationship between liver cancer and abnormal expression of miRNA, such as miR-1247-3p [16], miR-204 [17], and miR-498 [18], which indicates that abnormally expressed miRNA plays an important role in liver cancer. A previous study reported that miRNAs are involved in tumor transformation of liver cancer stem cells, including miR-889 [19]. Thus, it would be of interest to investigate the clinical significance and biological function of abnormally expressed miR-889 in liver cancer.

In this paper, we mainly study the abnormal expression of miR-889 in hepatocellular carcinoma cells and tissues, and the relationship between the abnormal expression of miR-889 and clinicopathological features was analyzed. We also investigated the overall survival and prognostic significance of liver cancer patients with high expression of miR-889. To further study the effects of miR-889 mimic, mimic negative control (mimic NC), miR-889 inhibitor or inhibitor NC on proliferation, migration, and invasion of hepatoma cells.

## Materials and methods

### Patients and tissue samples

A total of 113 fresh specimens of HCC and para-cancerous benign tissue were obtained from January 2010 and June 2015, among 113 patients who underwent partial or total hepatectomy in the Harbin Medical University Cancer Hospital. The fresh specimens obtained by surgery were immediately placed in liquid nitrogen and stored at -80℃ for future use. All the samples were confirmed by pathology, and all the patients did not receive radiotherapy, chemotherapy, biological therapy, or other relevant treatments before surgery. The clinical characteristics of all patients were collected and summarized in Table 1. All the included patients signed informed consent. This study was approved by the Ethics Committee of Harbin Medical University Cancer Hospital. A 5-year follow-up was conducted to obtain the prognosis of the patients.

**Table 1:** Association between miR-889 expression and different clinical characteristics of patients with hepatocellular carcinoma

Clinical characteristics	Cases	miR-889 expression		*P* values
	*n* = 113	Low (*n* = 56)	High (*n* = 57)	
Age				0.782
≥ 60	60	29	31	
< 60	53	27	26	
Gender				0.382
Male	66	35	31	
Female	47	21	26	
Tumor size (cm)				0.953
≥ 5	32	16	16	
< 5	81	40	41	
TNM stage				0.022
I-II	73	42	31	
III-IV	40	14	26	
HBV infection				0.006
Negative	46	30	16	
positive	67	26	41	
Cirrhosis				0.029
Negative	47	29	18	
positive	66	27	39	
Differentiation				0.127
Well-Moderate	75	41	34	
Poor	38	15	23	

*HBV* infection: Hepatitis B virus infection

### Cell culture and transfection

HCC cell lines HuH7, HCCLM3, MHCC97-H, HepG2, and normal liver cells (THLE-2) were all purchased from the Institute of Cell Research, Chinese Academy of Sciences (Shanghai, China). All cell lines were cultured in RPMI1640 culture medium (HyClone, Logan, UT, USA) supplemented with 10 % FBS (HyClone) and placed in a humidified incubator with 5 % CO_2_ at 37℃. The cells were observed daily and the culture medium was changed. Cells were passed on when they reach 80 % of the degree of fusion.

To investigate the expression level of miR-889 in cell lines, cells were transfected with miR-889 mimic (5’-UUAAUAUCGGACAACCAUUGU-3’), mimic negative control (mimic NC; 5’-UUCUCCGAACGUGUCACGUTT-3’), miR-889 inhibitor (5’-ACAAUGGUUGUCCGAUAUUAA-3’), and inhibitor NC (5’-CAGUACUUUUGUGUAGUACAA-3’) (GenePharma; Shanghai, China) using Lipofectamine 3000 (Thermo Fisher Scientific, Waltham, MA, USA). Untransfected cells served as a blank control group.

### RNA extraction and quantitative real-time PCR

The total RNA of HCC tissues or cells was extracted using TRIzol reagent (Invitrogen, Carlsbad, CA, USA), and the RNA quality and quantity were verified with a NanoDrop 2000 (Thermo Fisher Scientific). Reverse transcription was performed to obtain cDNA using the TaqMan miRNA reverse transcription kit (Thermo Fisher Scientific). The expression levels of miR-889 were determined by quantitative real-time PCR (qRT-PCR). qRT-PCR was performed with TaqMan miRNA quantitative PCR kit (Thermo Fisher Scientific) according to the kit instructions on an ABI 7500 PCR System. The sequences of the PCR primers used were as follows: miR-889 forward, 5’-GCCGAGTTAATATCGGACAAC-3’, reverse, 5’- CTCAACTGGTGTCGTGGA-3’; U6 forward, 5’-CTCGCTTCGGCAGCACA-3’ and reverse, 5’-AACGCTTCACGAATTTGCGT-3’. The relative expression levels of miR-889 were calculated by the 2^−ΔΔCT^ method. Repeat the experiment at least 3 times.

### Cell proliferation assay

For the cell proliferation assay, 5 × 10^3^ cells were plated in 96-well plates. Cell growth was determined by using a Cell Counting Kit-8 (CCK-8; Beyotime, Shanghai, China). 10 µL of CKK-8 solution (Dojindo, Kumamoto, Japan) was added to each well and incubated at 37 °C in 5 % CO_2_ for 2 h. The absorbance (OD) at 450 nm was measured, and the experiment was repeated 3 times.

### Cell migration and invasion assays

After transfection, the migration and invasion assays of HCC cells were conducted using a 24-well Transwell plate (8 μm; BD Biosciences, San Jose, CA). MHCC-97 H and HCCLM3 cells were suspended in 100 µL serum-free medium and placed in the upper chambers of the Transwell and incubated at 37 °C for 72 h for the cell migration and invasion assays. The main difference between the assays of cell migration and invasion is that cell migration Transwell chambers without Matrigel (BD Biosciences, San Jose, CA, USA), but the invasion assay has Matrigel. The cell density was 5 × 10^4^, and a normal cell culture medium containing fetal bovine serum was added to the lower layer of the chamber. After continuous culture for 24 h, the lower cells were fixed and stained for 30 min and 5 random fields were randomly selected for counting and statistics in each well.

### Statistical analysis

Statistical Product and Service Solutions (SPSS) 19.0 software (IBM, Armonk, NY, USA) and GraphPad 5.0 (GraphPad Software, Inc., La Jolla, CA, USA) were used for statistical analysis of the experimental data. The experimental results were expressed as mean ± standard deviation. A two-tailed t-test was used to compare the means between two sets, and one-way analysis of variance was used to compare the means among three groups. The overall survival was analyzed by the Kaplan-Meier method, and the multivariate survival analysis was performed by Cox regression. *P* < 0.05 was considered statistically significant.

## Results

### Expression of miR-889 in HCC tissues and cell lines

**Fig. 1:**
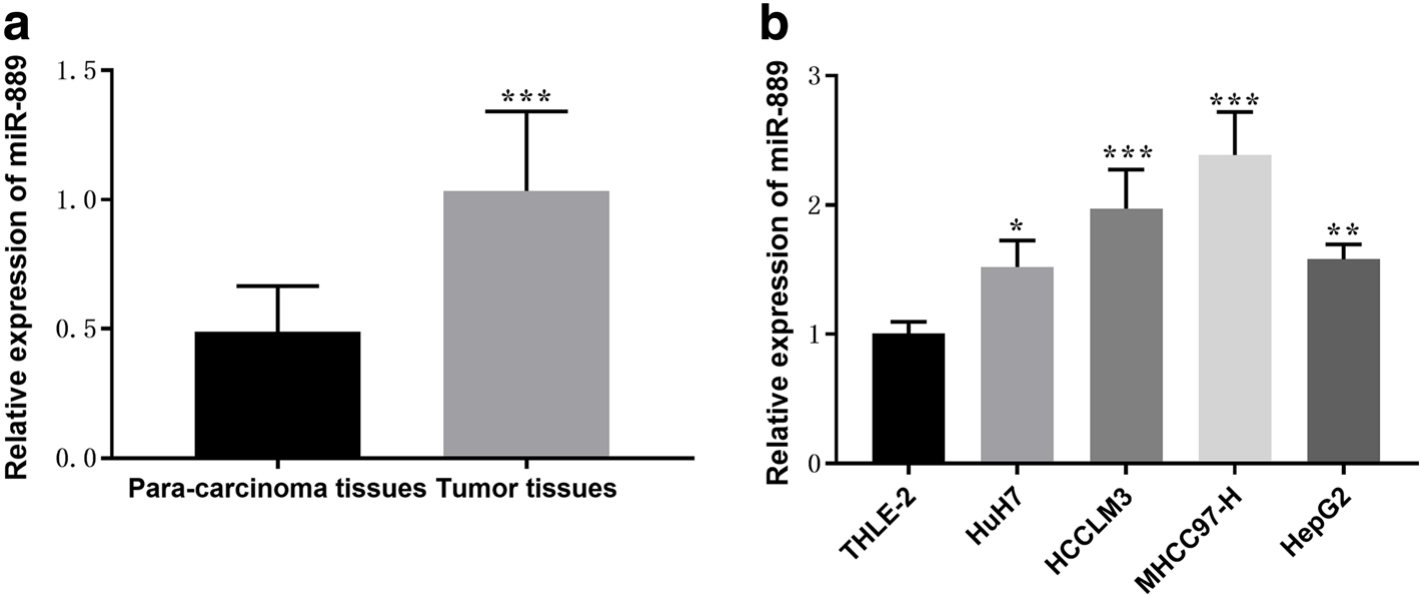
The expression of miR-889 was increased in HCC tissues and cell lines. (**a**) Expression levels of miR-889 were examined in all 113 pairs of HCC tissue samples (mean ± SD: 1.034 ± 0.3066). (**b**) Expression levels of miR-889 were detected in 4 HCC cell lines. ***P < 0.001

The expression of miR-889 in HCC tissues was detected by qRT-PCR. The results showed that the expression of miR-889 in HCC tissues was significantly higher than that in adjacent tissues (*P* < 0.001, Fig. 1 A). Meanwhile, the expression of miR-889 in four HCC cell lines (HuH7, HCCLM3, MHCC97-H, HepG2) was also higher than that in normal cell line (THLE-2, *P* < 0.05, Fig. 1B). Among the four HCC cell lines, MHCC97-H and HCCLM3 cell lines exhibited higher miR-889 expression than the othe two HCC cell lines, which were chosen for subsequent cellular experiments.

### Relationship between clinicopathological characteristics and miR-889 expression in HCC patients

According to the median expression level of miR-889 in 113 patients, the patients were divided into the high expression group (*n* = 57) and the low expression group (*n* = 56). Table 1 shows the relationship between the expression level of miR-889 and clinicopathological variables in HCC patients. The expression of miR-889 in HCC tissues is related to TNM staging, hepatitis B virus infection, and cirrhosis (*P* < 0.05), While the expression of miR-889 is not statistically significant with age, gender, tumor size, and differentiation (*P* > 0.05).

### Upregulation of miR-889 is associated with poor prognosis in HCC patients

**Fig. 2:**
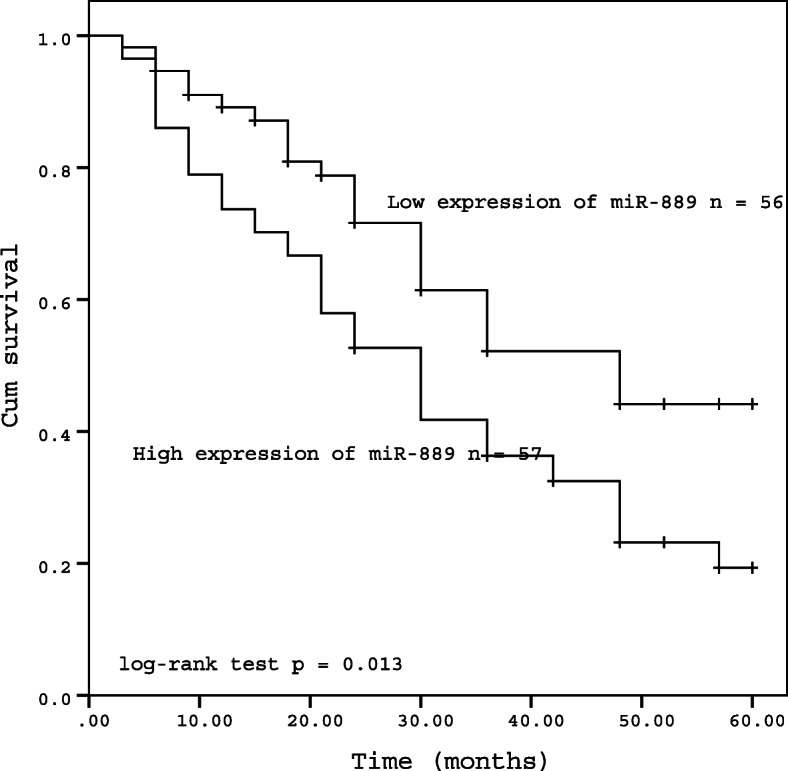
Kaplan-Meier curve of survival time in patients with HCC. (log-rank test *P* = 0.013)

Considering the significant correlation between miR-889 expression level and TNM staging, hepatitis B virus infection, and cirrhosis, we speculated that miR-889 might be an independent prognostic factor for HCC. To confirm this hypothesis, the Kaplan-Meier survival curve was used to analyze the relationship between miR-889 and the survival of patients. The results indicated that the overall survival of patients in the high expression group was significantly lower than that in the low expression group (*P* = 0.013, Fig. 2). The univariate Cox regression results (Table 1) showed that miR-889 expression (*P* = 0.020), TNM stage (*P* = 0.048), hepatitis B virus infection (*P* = 0.030), and cirrhosis (*P* = 0.006) were risk factors to the survival of patients. Furthermore, multivariate Cox proportional regression model indicated that miR-889 expression (*P* = 0.009) and TNM stage (*P* = 0.011) were independent prognostic factors for overall survival (shown in Table 2).

**Table 2:** Cox analysis of factors for survival of hepatocellular carcinoma patients

Variables	Univariate Cox analysis			Multivariate Cox analysis		
	HR	95 %CI	*P* value	HR	95 %CI	*P* value
miR-889	1.828	1.101–3.034	0.020	2.125	1.204–3.751	0.009
Age	1.062	0.654–1.725	0.808	1.263	0.759–2.102	0.369
Gender	0.975	0.599–1.587	0.919	1.118	0.663–1.886	0.676
Tumor size	1.511	0.837–2.726	0.171	1.756	0.953–3.235	0.071
TNM stage	1.718	1.005–2.937	0.048	2.190	1.198–4.004	0.011
HBV infection	1.769	1.058–2.959	0.030	1.622	0.899–2.926	0.108
Cirrhosis	2.098	1.239–3.553	0.006	1.605	0.929–2.774	0.090
Differentiation	1.423	0.868–2.333	0.162	1.597	0.924–2.758	0.093

*HBV* infection: Hepatitis B virus infection

### Upregulation of miR-889 promotes HCC cell proliferation, migration, and invasion

**Fig. 3:**
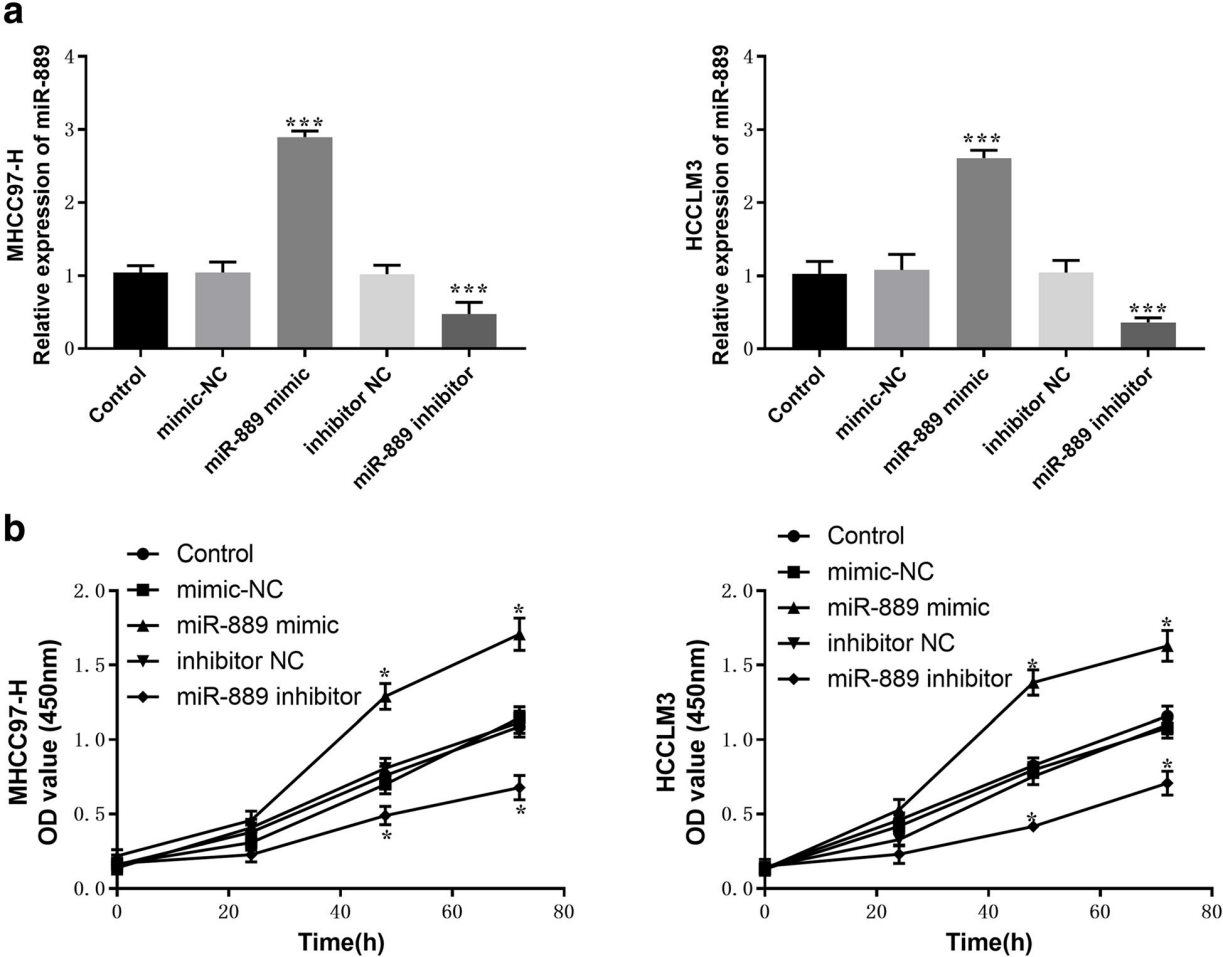
Effects of miR-889 expression levels on proliferation in MHCC97-H and HCCLM3 cells. **a** the expression level of miR-889 was verified by qRT-PCR after transient transfection with miR-889 mimic/inhibitor (or mimic/inhibitor NC). **b** The CCK-8 assay was performed to examine cell proliferation. *P < 0.05, ***P < 0.001

In order to study the role of miR-889 in the occurrence and development of HCC, cell proliferation, migration, and invasion experiments were carried out in vitro. MHCC97-H and HCCLM3 cells were transfected with miR-889 mimic, mimic NC, miR-889 inhibitor, and inhibitor NC. The qRT-PCR result shows that miR-889 mimic could significantly increase the expression of miR-889, while miR-889 inhibitor could decrease the expression of miR-889, compared with control (*P* < 0.001, Fig. 3 A). A CCK-8 assay was used to determine the effect of miR-889 on the proliferation of HCC cells in the study. The results showed that the high expression of miR-889 could promote the proliferation of HCC cells, while the low expression of miR-889 could inhibit the proliferation of liver cancer cells, compared with the control group (*P* < 0.05, Fig. 3B).

**Fig. 4:**
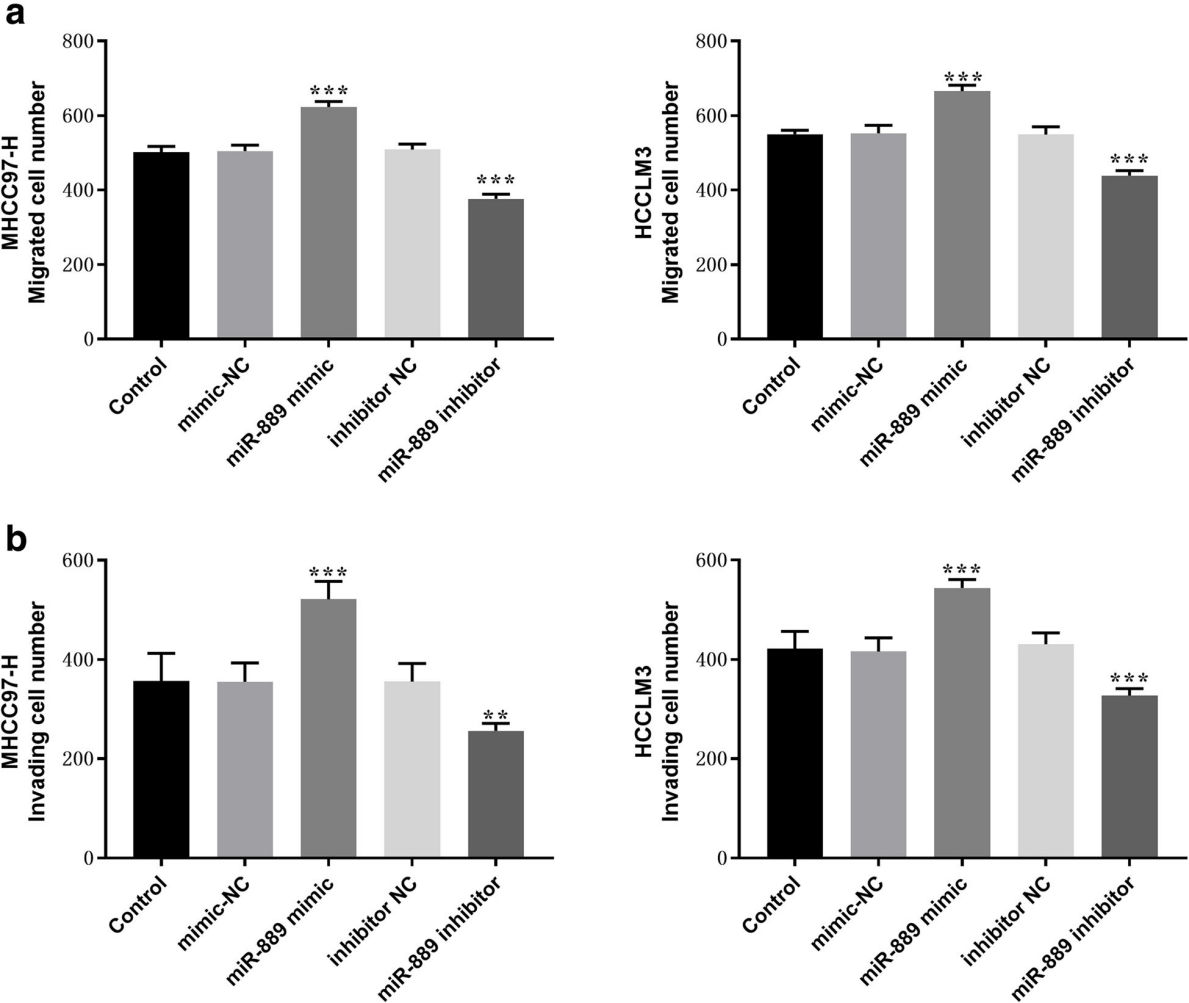
Effects of miR-889 on cell migration and invasion abilities in MHCC97-H and HCCLM3 cells. (**a**) Cell migration and (**b**) invasion abilities were assessed with Transwell assay. **P < 0.01, ***P < 0.001

Transwell assay was used to detect the migration and invasion of MHCC97-H and HCCLM3 cells. The results showed that overexpression of miR-889 significantly increased the migration and invasion ability of HCC cells compared with the blank control group and NC group (*P* < 0.01, Fig. 4 A − B).

## Discussion

HCC is one of the most common pathological types of primary liver cancer [20, 21]. It has the characteristics of rapid invasive growth and early distant metastasis. About 60 − 80 % of HCC patients have lost the opportunity of radical surgery at the first visit. According to epidemiological survey data, HCC is the second and third leading cause of death in men and women respectively in China [22]. Therefore, the search for new and effective molecular markers and biotherapy targets for HCC has become an important way to improve the level of diagnosis and treatment of HCC in China [23, 24].

As a class of endogenous small RNA molecules [25], miRNAs can combine with incomplete mRNA sequences of their target genes to form hairpin-like structures in mammals, which can enhance or inhibit the translation process, thus up-regulating or down-regulating the expression level of target genes and realizing the role of regulatory genes [26, 27]. miR-889 is a new miRNA that has been found to have multidirectional tumor regulation in recent years.

In the present study, we found that miR-889 was up-regulated in HCC cell lines, and its level in HCC was significantly higher than that in adjacent tissues and normal liver tissues. The abnormally low expression of miR-889 has also been studied in other cancers. For example, in cervical cancer, reduced miR-889 expression was significantly associated with the International Federation of Gynecology and Obstetrics cancer staging and with lymph node metastasis, suggesting that downregulated miR-889 expression may be linked with the development of cervical cancer [28]. Moreover, the majority of patients with high miR-889 expression exhibited advanced TNM stage, positive hepatitis B virus infection and cirrhosis, miR-889 was related to poor prognosis and early recurrence in HCC. The above results suggest that up-regulation of miR-889 may be involved in HCC progression. The previous report of a comprehensive approach of HCC, miR-889 was not listed among the miRNAs reported as poor prognosis factors for HCC [29]. In the present study, Kaplan-Meier and multivariate Cox analysis results showed that patients with the high expression of miR-889 might have a poor prognosis, suggesting that the expression of miR-889 can be considered as a potential predictor of HCC, which is an interesting finding. Similar findings have also been reported in osteosarcoma, HSA-miR − 889-3p has been identified as highly expressed and can act as an independent prognostic factor for osteosarcoma [30].

Since miR-889 is highly expressed in highly invasive HCC cell lines, we speculate that miR-889 may have the function of carcinogenic factors. To verify this, the effects of miR-889 on HCC cell proliferation, migration, and invasion were assessed. The results showed that overexpression of miR-889 significantly increased cell proliferation, migration, and invasion in transfected HCC cell lines, while inhibition of miR-889 expression decreased these cell behaviors. Overall, these results suggested that miR-889 may play a promoting role in HCC. Previous studies demonstrated that miR-889 could regulate the tumor cellular behaviors by targeting serval target genes, such as KLF9 [31], SIX1 [32], DAB2IP [33], TAB1 [34], FGFR2 [28], TWEAK [35]. For instance, miR-889 expression was significantly up-regulated in non-small cell lung cancer cell lines and tissues, and miR-889 may play a potential therapeutic role in non-small cell lung cancer by targeting KLF9 to control the proliferation and migration of non-small cell lung cancer [31]. In colorectal cancer, miR-889 may be involved in controlling the proliferation of CRC by regulating DAB2IP, which provides potential and potential therapeutic targets for colorectal cancer [33]. Both KLF9 and DAB2IP were reported to be tumor suppressors in many tumors [36–38]. A previous study indicated that KLF9 suppresses the tumor cell growth in HCC by positively regulating p53 expression [39]. Besides, low expression of DAB2IP contributes to malignant development (including proliferation and invasion) and poor prognosis in HCC [40]. Combination of present data and previous study results, we hypothesized that miR-889 may play an oncogenic role in HCC by targeting KLF9 or DAB2IP. However, the detailed target genes of miR-889 remain need to be confirmed in the future study. Besides, the molecular mechanisms of miR-889 in HCC remain unclear. In future work, we will carry out further study in this aspect. What’s more, considering the background liver disease may have affect to the miRNA expression, we will take into account the background of liver disease in further studies.

Taken together, miR-889 has higher expression levels in HCC cell lines and tissues than in adjacent tissues. Further studies show that the overexpression of miR-889 is related to the poor prognosis and shorter survival time of HCC. In addition, overexpression of miR-889 also significantly increases cell proliferation, migration, and invasion in transfected HCC cell lines. Therefore, these data suggest that miR-889 may be involved in the occurrence and development of HCC as a novel oncogene. The abnormal expression of miR-889 has a significant impact on the survival and prognosis of HCC patients. miR-889 provides a promising target for the prognosis and treatment of HCC.

## Data Availability

The datasets used and/or analysed during the current study are available from the corresponding author on reasonable request.
